# Adalimumab ameliorates memory impairments and neuroinflammation in chronic cerebral hypoperfusion rats

**DOI:** 10.18632/aging.203009

**Published:** 2021-05-24

**Authors:** Jing-Jing Xu, Si Guo, Rui Xue, Lin Xiao, Jun-Na Kou, Yu-Qiong Liu, Jun-Ya Han, Jing-Jie Fu, Na Wei

**Affiliations:** 1 Department of Pathology, The First Affiliated Hospital of Zhengzhou University, Zhengzhou 450002, People’s Republic of China; 2 Henan Key Laboratory of Tumor Pathology, Zhengzhou 450002, People’s Republic of China; 3 Department of Pathology, School of Basic Medicine, Zhengzhou University, Zhengzhou 450002, People’s Republic of China; 4 Department of Medical Laboratory, Henan Provincial People’s Hospital, Zhengzhou, Henan 450003, People’s Republic of China; 5 Department of Medical Laboratory of Central China Fuwai Hospital, Zhengzhou, Henan 450003, People’s Republic of China; 6 Central China Fuwai Hospital of Zhengzhou University, Zhengzhou, Henan 450003, People’s Republic of China; 7 Medical Research Center, The First Affiliated Hospital of Zhengzhou University, Zhengzhou 450002, People’s Republic of China; 8 Department of Pathology, People’s Hospital of Zhengzhou, Zhengzhou 450000, People’s Republic of China

**Keywords:** adalimumab, vascular dementia, neuroinflammation

## Abstract

Vascular dementia (VaD) is the second most common type of dementia worldwide. Although there are five FDA-approved drugs for the treatment of Alzheimer’s disease (AD), none of them have been applied to treat VaD. Adalimumab is a TNF-α inhibitor that is used for the treatment of autoimmune diseases such as rheumatoid arthritis. In a recent retrospective case-control study, the application of adalimumab for rheumatoid or psoriasis was shown to decrease the risk of AD. However, whether adalimumab can be used for the treatment of VaD is not clear. In this study, we used 2VO surgery to generate a VaD rat model and treated the rats with adalimumab or vehicle. We demonstrated that VaD rats treated with adalimumab exhibited significant improvements in memory. In addition, adalimumab treatment significantly alleviated neuronal loss in the hippocampi of VaD rats. Moreover, adalimumab significantly reduced microglial activation and reversed M1/M2 polarization in VaD rats. Furthermore, adalimumab treatment suppressed the activity of NF-κB, an important neuroinflammatory transcription factor. Finally, adalimumab displayed a protective role against oxidative stress in VaD rats. Our results indicate that adalimumab may be applied for the treatment of human patients with VaD.

## INTRODUCTION

Dementia is one of the most prevalent neuropsychiatric disorders in aged people and causes a long-term or gradual decline in learning/memory and some emotional problems. With the rapid increase of the aging population, the prevalence of dementia is increasing year to year. Vascular dementia (VaD) is the second most common type of dementia, accounting for approximately 20% of all dementia cases. The pathogenesis of vascular dementia is poorly understood, so there is currently no effective therapeutic strategy for VaD. None of the four FDA-approved medications for Alzheimer's disease medications, i.e., donepezil, galantamine, rivastigmine and memantine, are used to treat vascular dementia, but these drugs may be used in people with a combination of vascular dementia and Alzheimer's disease. Thus, it is important to develop a new approach for the treatment of VaD based on underlying mechanistic studies.

Although the detailed pathogenesis is not clear, VaD has been suggested proven to be associated with different cerebral vascular events, including ischemic, hemorrhagic, anoxic, and hypoxic insults. In response to such acute or chronic vascular accidents, endothelial cells are abnormally activated and lead to deregulation of the blood-brain barrier and brain edema or damage, which results in the activation of microglia, astrocytes or other glial cells to initiate neuroinflammation. In the brains of VaD patients, the neuroinflammation process is significantly activated. Toll-like receptor 4 (TLR4), a transmembrane protein expressed on microglia and astrocytes, is more highly expressed in the VaD brain than in the healthy brain [1]. The expression of TLR4 can be activated by hypoxia [2] and amyloid-β (Aβ) [3, 4], which have also been observed in the VaD brain [1]. Importantly, Aβ is able to bind with TLR4, resulting in the augmentation of the levels of multiple proinflammatory cytokine factors [5]. Among these factors, TNF-α is particular of interest. Although both astrocytes and neurons can be stimulated and produce TNF-α during inflammation [6], activated microglia are thought to be the dominant source of this cytokine [7] to mediate the inflammatory response in the central nervous system [8]. It is known that TNF-α is able to directly stimulate the acute phase reaction and indirectly mediate the chronic inflammatory reaction via its known downstream pathological cascades. Moreover, an increase in TNF-α levels can also promote the generation of other proinflammatory cytokines (nitric oxide (NO), IL-1β and IL-6) or reactive oxygen species (ROS) to induce demyelination, neuronal degeneration and excitotoxicity [9]. Therefore, targeting TNF-α should be a promising therapeutic approach for neuroinflammatory disorders.

Indeed, due to the well-known roles of TNF-α in the stimulation of Aβ overproduction and tauopathy and AD-like cognitive dysfunction, anti-TNF-α treatment has been shown to be effective in rescuing cognitive impairments and pathological changes in AD [10]. In another independent study, treatment with adalimumab was shown to improve cognitive deficits and alleviate AD pathology in an animal model of AD [11]. Given the similarity of the neuroinflammatory process and neuronal death-related pathology in AD and VaD, we raised the question of whether anti-TNF-α treatment can ameliorate memory impairments and microglia-based neuroinflammation in a VaD model. In the current study, we generated a VaD rat model by performing two-vessel occlusion (2VO) surgery, treated VaD rats with adalimumab or vehicle for 9 weeks and assessed memory, microglial subtypes, neuronal death, ROS levels and downstream signaling pathways.

## MATERIALS AND METHODS

### Animals and treatment

**Figure 1:**
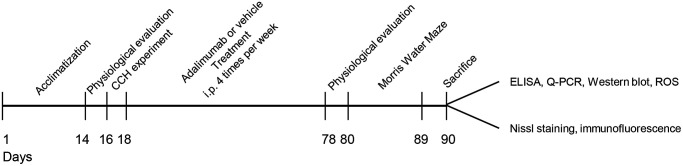
**Experimental diagram of the current study.** A total of 40 rats were first acclimated for 2 weeks, and then physiological parameters were evaluated. After being subjected to 2VO or sham surgery, the rats were treated with adalimumab or vehicle for 9 weeks (four times a week). At the end of drug injection, physiological parameters were evaluated again. Then, the rats were subjected to the Morris water maze test and sacrificed after the behavior test. Samples were collected for biochemical and morphological experiments accordingly.

A total of 40 SD rats (3 months; weight, 220–250 g) were purchased from Zhengzhou University Laboratory Animal Center. All the animals were housed under a 12-h light-dark cycle (lights on 8 a.m.) at 25°C and 60 ± 10% humidity and given *ad libitum* access to food and water. The rats were divided into four groups: the sham operation and vehicle treatment (sham) group, the bilateral common carotid artery ligation (2VO) surgery and vehicle treatment (chronic cerebral hypoperfusion, CCH) group; the 2VO surgery and ADA treatment (CCH+ADA) group and the sham operation and ADA treatment (ADA) group. There were 10 rats in each group, and the rats were divided in a double-blinded manner. The experimental procedures (Figure 1) were performed in accordance with the guidelines of the National Institutes of Health Guide for the Care and Use of Laboratory Animals and approved by the Animal Ethics Committee of the Medical School of Zhengzhou University.

### Drugs, antibodies and kits

Adalimumab (Humira) was purchased from AbbVie Inc. (North Chicago, IL, USA). It was first dissolved in the N.S at a final concentration of 1 mg/mL and then injected intraperitoneally (i.p.) at a dose of 5 mg/kg four times a week for 9 weeks [12]. Anti-Iba1 (ab178847, 1:200 for immunofluorescence), anti-nuclear factor kappa B (NF-kB) p65 (p65, ab16502, 1:1000 for western blotting), anti-phospho-Ser536-NF-kB p65 (p-p65, ab-28856, 1:800 for western blotting), anti-STAT3 (ab68153, 1:1000 for western blotting) and anti-β-actin (ab6276, 1:1000 for western blotting) antibodies were purchased from Abcam Inc. (Shanghai, China). Commercial kits for detecting the inflammatory factors TNFα (kt30484), IL-6 (kt30490), IL-12 (kt30428), IFNγ (kt77521), IL-1β (kt45331), IL-10 (kt30495) and IL-4 (kt30491) were purchased from MSK Company (Wuhan, China). ELISA kits for measuring SOD (A001-3-2), MDA (A003-1-2), GSH-Px (A005-1-2), and CAT (A007-1-1) levels were purchased from Nanjing Jianchen Company (Nanjing, China).

### 2VO surgery

The rats were administered a mixture of ketamine (80 mg/kg) and xylazine (10 mg/kg) intraperitoneally for anesthetization. Throughout the whole surgical operation, the room temperature was kept at 25°C, and the rats were placed on a 37° heating pad. Then, the common carotid arteries on both sides were gently separated and doubly ligated by using 4-0 silk sutures just under the carotid bifurcation. The sham group also underwent surgery but without not ligation. To verify that 2VO surgery was successful, a laser Doppler system was used to evaluate blood flow, and a reduction to a level 70% less than normal was considered to indicate success [13, 14].

### Morris water maze

To evaluate spatial learning and memory, the Morris water maze test was employed as described previously [15]. The water maze was composed of a circular pool with a diameter of 180 cm filled with water at 20–22°C. All the rats were first habituated to the experimental room for 1 hour before the formal experiment. For the first 3 days, the rats were trained to find a visible platform to evaluate vision. Then, the platform was hidden 0.5 cm below the surface of opaque water for a 5-day (four trials a day) training phase. In each trial, the mice were given a maximum of 60 s to find the hidden platform and were allowed to stay on it for 30 s. The trial ended when the mouse reached the platform. Mice that failed to locate the platform within 60 s were guided to the platform by the experimenter, and their data were discarded. After one day of rest, the platform as removed for the probe trial. In each trial, the mice were placed in the pool in the quadrant opposite of where the platform was located. An overhead camera was used to capture the movement of each rat every day. The escape latency and swimming traces were recorded. In each trial, the rat was placed into the pool from one of four possible locations (randomly ordered). The escape latency was defined as the time required to reach the escape platform. If the rat did not find the platform within 60 sec, it was gently guided by the observer to the platform, where it remained for 30 sec. In the probe trial task, the swimming path, percentage of time spent in the target quadrant, number of platform area crossings, and latency to first cross the platform were recorded and calculated by computer software.

### Nissl staining

The rats were anesthetized with ketamine and xylazine and perfused with 4% paraformaldehyde in 0.1 mol/L phosphate buffer solution (pH 7.4). The whole brains were removed and postfixed in 4% paraformaldehyde at 4°C for an additional 24 h. After they were dehydrated in 30% and 40% sucrose until they sank, the brains were rapidly frozen in isopentane, and 25-μm thick coronal sections were cut on a cryostat (CM 1950, Leica, Heidelberger, Germany). All the sections were used for Nissl staining with 0.1% cresyl violet (Sigma, St Louis, MO, USA) to evaluate neuronal damage in the hippocampus. For cell counting, NIH ImageJ software was used as previously reported [13].

### Immunofluorescence

The immunofluorescence assay was performed according to a previous report [16]. Frozen slices (30 μm) were rinsed with PBS (pH 7.4) for 5 min three times each and then incubated with 0.5% Triton in PBS for half an hour. After blocking with 0.5% BSA for 1 h, the cells were incubated with an anti-Iba1 antibody overnight at 4°C. After rinsing with PBS, the slices were incubated with anti-rabbit IgG (H+L) (Alexa Fluor 488-conjugated; Life Technologies, USA) at room temperature for 1 h in the dark. After staining with DAPI for 5 min at room temperature, the slices were photographed using a confocal laser scanning microscope (LSM780, Carl Zeiss, Oberkochen, Germany).

### Western blotting

Hippocampal tissues were mixed and homogenized with sample buffer (pH 7.6; 50 mM Tris-HCl, 10 mM dithiothreitol, 2% sodium dodecyl sulfate, 10% glycerol, and 0.2% bromophenol blue) on ice and boiled for 10 minutes. Thirty micrograms of protein was heated at 95°C for 5 minutes and then cooled on ice for 5 minutes. The protein was fractionated on a 4–12% SDS-PAGE Bis-Tris gel using an SDS-PAGE system (Bio-Rad, CA, USA). The proteins were transferred onto nitrocellulose membranes (Whatman, Kent, UK). Following blocking in 5% nonfat milk in 0.01% Tween PBS (PBST), the membranes were incubated with optimally diluted primary antibodies overnight at 4°C. Following washing with PBST, the membranes were incubated with the appropriate anti-mouse or anti-rabbit secondary antibody diluted 1:10,000 for 1 h at room temperature and visualized using the Odyssey Infrared Imaging System. The protein bands were quantitatively analyzed by Kodak Digital Science 1D software (Eastman Kodak Company, New Haven, CT, USA).

### NF-κB activity assay

For the measurement of NF-κB activity, the nuclear fraction was first extracted by using an NE-PER nuclear extraction kit (Thermo Fisher Scientific, Waltham, MA, United States) and then subjected to an NF-κB activity assay (Abcam, ab133112, Cambridge, MA, United States).

### Oxidative stress analysis

Superoxide dismutase (SOD), malondialdehyde (MDA), glutathione peroxidase (GSH-Px) and catalase (CAT) levels were measured according to the manufacturer’s instructions.

### Statistical analysis

Statistical analysis was performed using SPSS 20.0. The differences between the groups were analyzed by ANOVA followed by Dunnett’s *post hoc* test. For a single comparison, the significance of the difference was determined by *t*-test. A value of *p* < 0.05 was considered statistically significant.

## RESULTS

### Adalimumab did not affect physiological parameters in rats

Because physiological status, such as body weight, temperature, heart rate and respiratory rate, can affect the final outcomes of behavioral and biochemical tests, we first measured these physiological parameters in rats given different treatments. We found that there was no significant difference among the four groups in any of the physiological parameters. These data suggest that neither 2VO surgery nor ADA treatment altered the general physiological functions of the rats (Table 1).

**Table 1:** Both the 2VO surgery and adalimumab treatment didn’t alter the physiological parameters.

	**Sham**	**CCH**	**CCH + ADA**	**ADA**
*n*	10	10	10	10
BW-Pre	248.52 ± 13.15	243.30 ± 12.12	247.88 ± 13.79	243.78 ± 21.95
BW-Post	304.84 ± 17.09	302.68 ± 11.17	306.48 ± 10.14	306.09 ± 12.12
RF	84.10 ± 4.43	82.40 ± 4.60	81.80 ± 4.00	84.80 ± 3.58
HR	400.00 ± 13.30	404.60 ± 17.24	412.00 ± 11.27	409.10 ± 11.42
Temp	36.34 ± 0.33	36.70 ± 0.38	36.48 ± 0.25	36.54 ± 0.38

All the data are expressed as the mean ± S.D. Abbreviations: BW-Pre: body weight before the surgery; BW-Post: body weight after all the treatment; RF: respiratory frequency; HR: heart rate; Temp: body temperature; CCH: chronic cerebral hypoperfusion; ADA: Adalimumab treatment.

### Adalimumab rescued memory impairments in CCH rats

**Figure 2:**
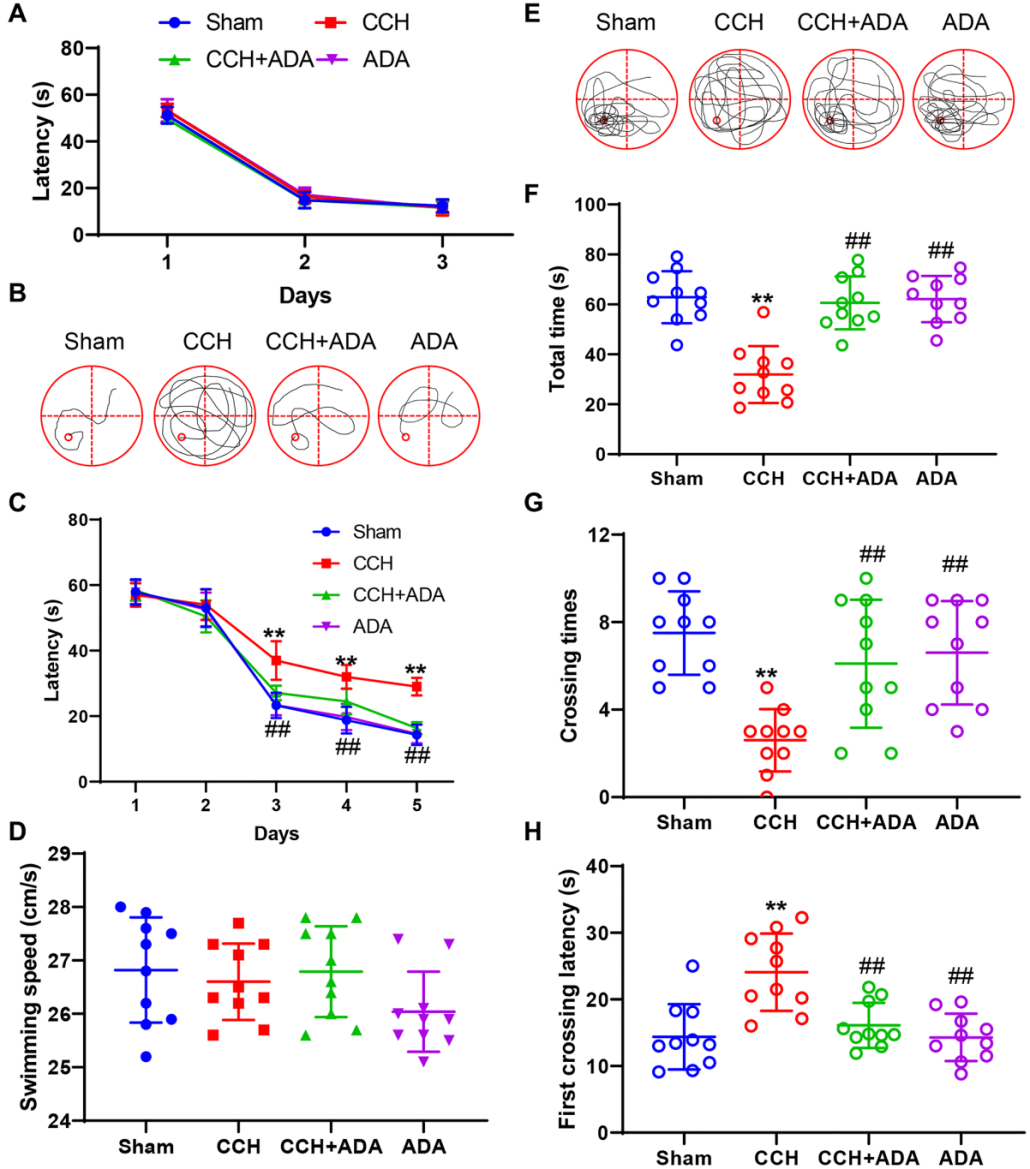
**ADA rescued memory impairments in CCH rats.** (**A**) The escape latency to find the visible platform in the first 3 days. (**B**) Representative traces of rats given different treatments on day 5 of the training phase. (**C**) Escape latency to find the hidden platform on days 1-5 of the training phase. (**D**) The swimming speeds of the different groups in the training phase. (**E**) Representative traces of rats given different treatments in the probe trial. (**F**) The total amount of time spent in the target quadrant in the probe trial. (**G**) The number of platform region crossings in the probe trial. (**H**) The latency to first platform region crossing in the probe trial. All values are expressed as the mean ± SEM (*n* = 10). ^**^p < 0.01 vs. the sham group; ^##^*p* < 0.01 vs. the CCH group.

To elucidate the potential protective role of ADA treatment in CCH rats, we used the Morris water maze test to evaluate the memory retention ability of the four groups. We first performed a three-day visible platform test to determine whether 2VO surgery or ADA treatment affected vision. As shown in Figure 2A, the learning traces of the four groups showed high similarity, indicating that vision was intact in all groups. Then, we subjected the rats to the hidden platform task. We found that after 5 days of training, the search strategy of the CCH rats was still random, while sham rats, CCH+ADA rats and ADA rats displayed a straightforward or target-based style (Figure 2B). By analyzing latencies on the five days of the learning phase, we found that CCH rats showed a longer latency to find the platform than sham rats on the third day. Rats treated with ADA displayed a shorter latency than the CCH rats, and no difference in latency was found among the sham, CCH+ADA and ADA rats (Figure 2C). The discrepancies among the four groups were not due to locomotor disability because the swimming speeds of the rats in the four groups were comparable (Figure 2D). In the probe trial test, the rats in the CCH group showed a shorter total time, fewer crossings and a longer latency to first crossing than the rats in the sham group, while ADA treatment significantly reversed these abnormalities (Figure 2E–2H). These data strongly suggested that ADA treatment can rescue learning and memory impairments in CCH rats.

### Adalimumab rescued neuronal loss in CCH rats

**Figure 3:**
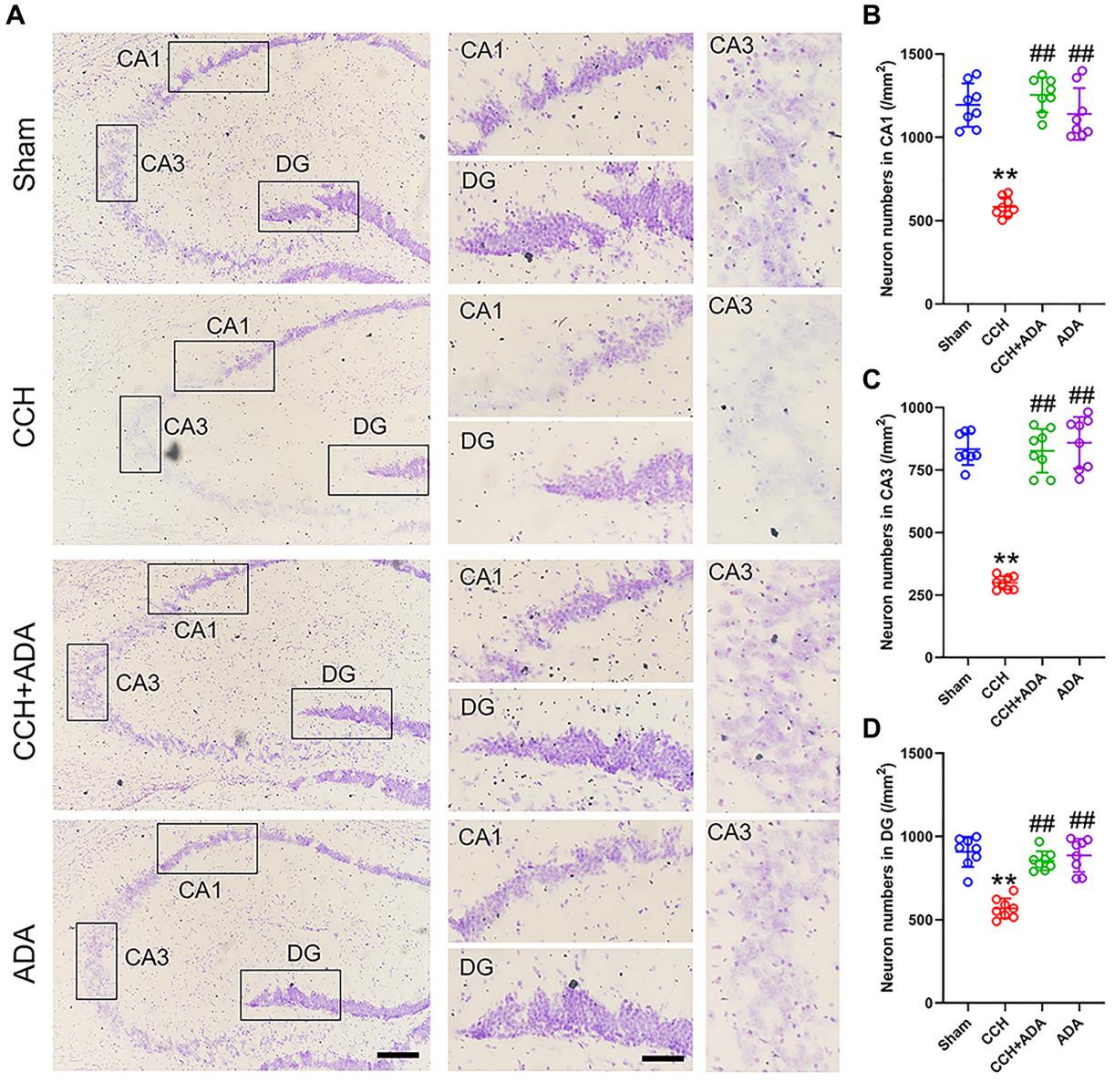
**ADA rescued neuronal loss in CCH rats.** (**A**) Representative images of Nissl staining for the different groups. Left: the whole hippocampus; Right: magnified images of the CA1, CA3 and DG regions. Bar = 100 μm for the left panel and 20 μm for the right panel. (**B**–**D**) Quantification of neuron number in the different groups in the CA1 (B), CA3 (C) and DG (D) regions. All values are expressed as the mean ± SEM (*n* = 8). ^**^p < 0.01 vs. the sham group; ^##^*p* < 0.01 vs. the CCH group.

Previous studies suggested that the degree of neuronal loss in the CA1 and CA3 regions positively correlates with the severity of memory impairment [17]. We thus performed Nissl staining to explore whether ADA administration could rescue neuronal loss in the hippocampi of CCH rats. We found that the neuron number in CCH rats was significantly reduced in the CA1, CA3 and DG regions, which was consistent with previous reports [18]. After treatment with ADA for 2 months, neuronal loss was apparently ameliorated (Figure 3A–3D). No difference was found between the ADA alone-treated group and the sham group.

### Adalimumab alleviated microglial activation and polarization in CCH rats

**Figure 4:**
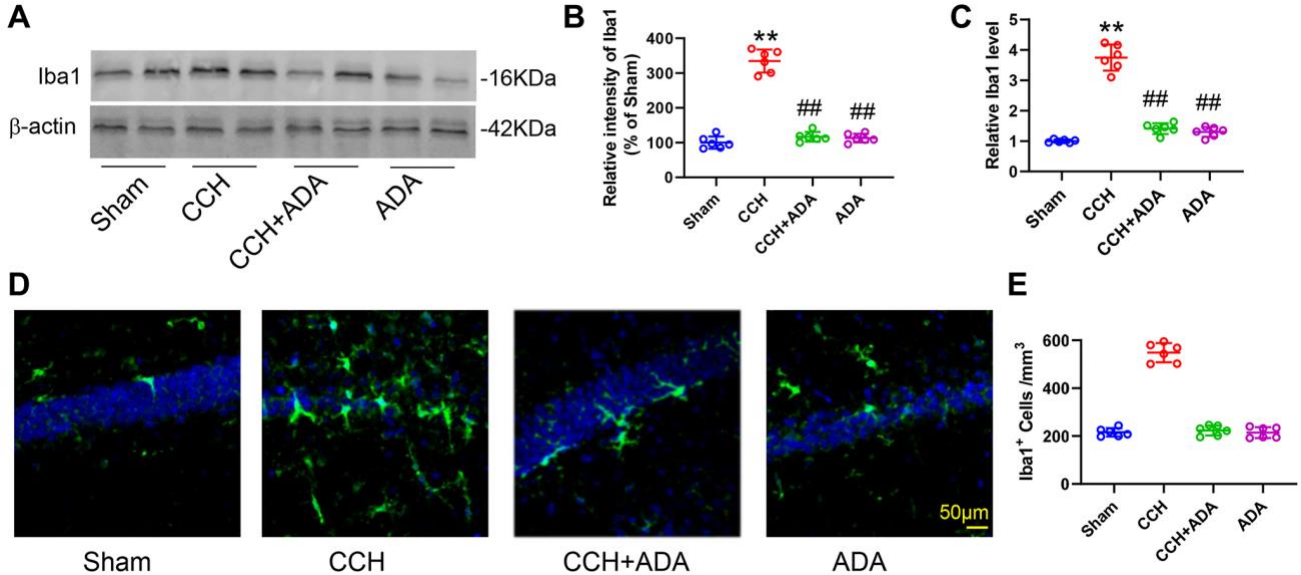
**ADA alleviated microglial activation in CCH rats.** (**A**) Representative blots of Iba1 in the different groups. β-Actin was used as loading control. (**B**) Quantification of the optical intensity of Iba1 in (A). (**C**) mRNA expression of Iba1 in the different groups. (**D**) Representative immunofluorescence images of Iba1 in the different groups. (**E**) Quantification of Iba1+ cells in (D). All values are expressed as the mean ± SEM (*n* = 6). ^**^p < 0.01 vs. the sham group; ^##^*p* < 0.01 vs. the CCH group.

Neuroinflammation, especially that mediated by activated microglia, has been suggested to play important roles in brain damage in CCH [19, 20]. We thus evaluated microglial activation and polarization in CCH rats treated with ADA. We found that CCH led to the elevation of Iba1 mRNA and protein levels and an increase in the number of Iba1-positive cells in the hippocampus (Figure 4A–4E). Administration of ADA reversed the activation of microglia induced by CCH.

**Figure 5:**
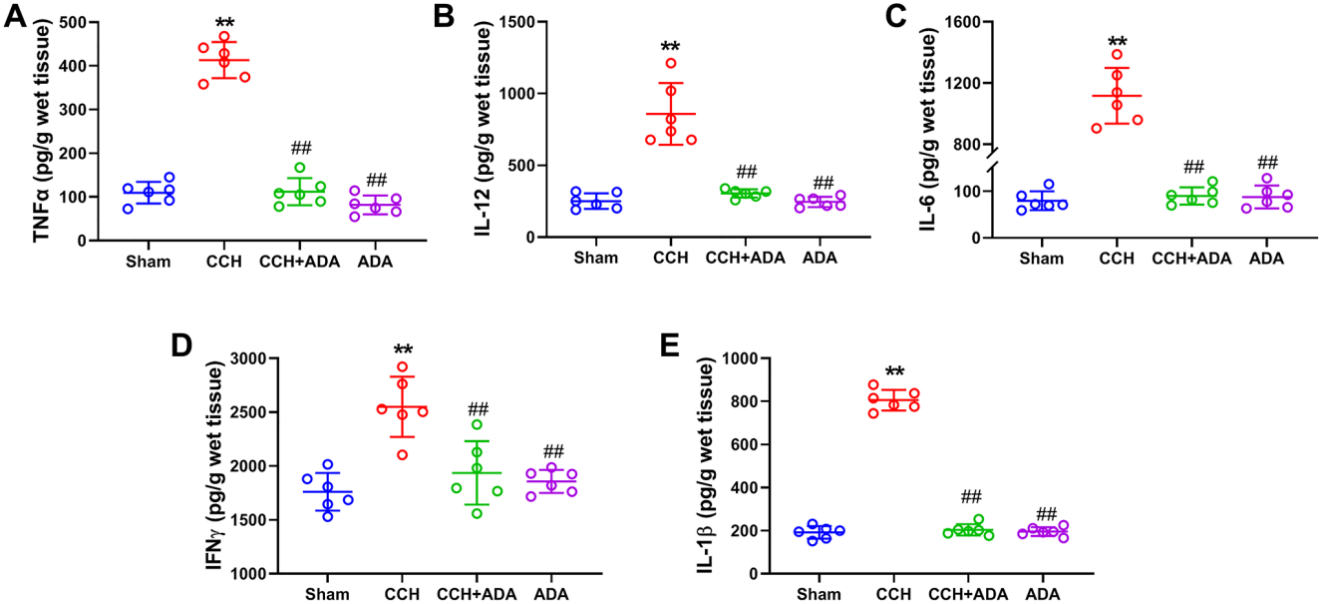
**ADA suppressed the expression of M1 markers.** The expression of the M1 markers TNFα (**A**), IL-12 (**B**), IL-6 (**C**), IFNγ (**D**) and IL-1β (**E**) in the different groups was evaluated by ELISA kits. All values are expressed as the mean ± SEM (*n* = 6). ^**^p < 0.01 vs. the sham group; ^##^*p* < 0.01 vs. the CCH group.

**Figure 6:**
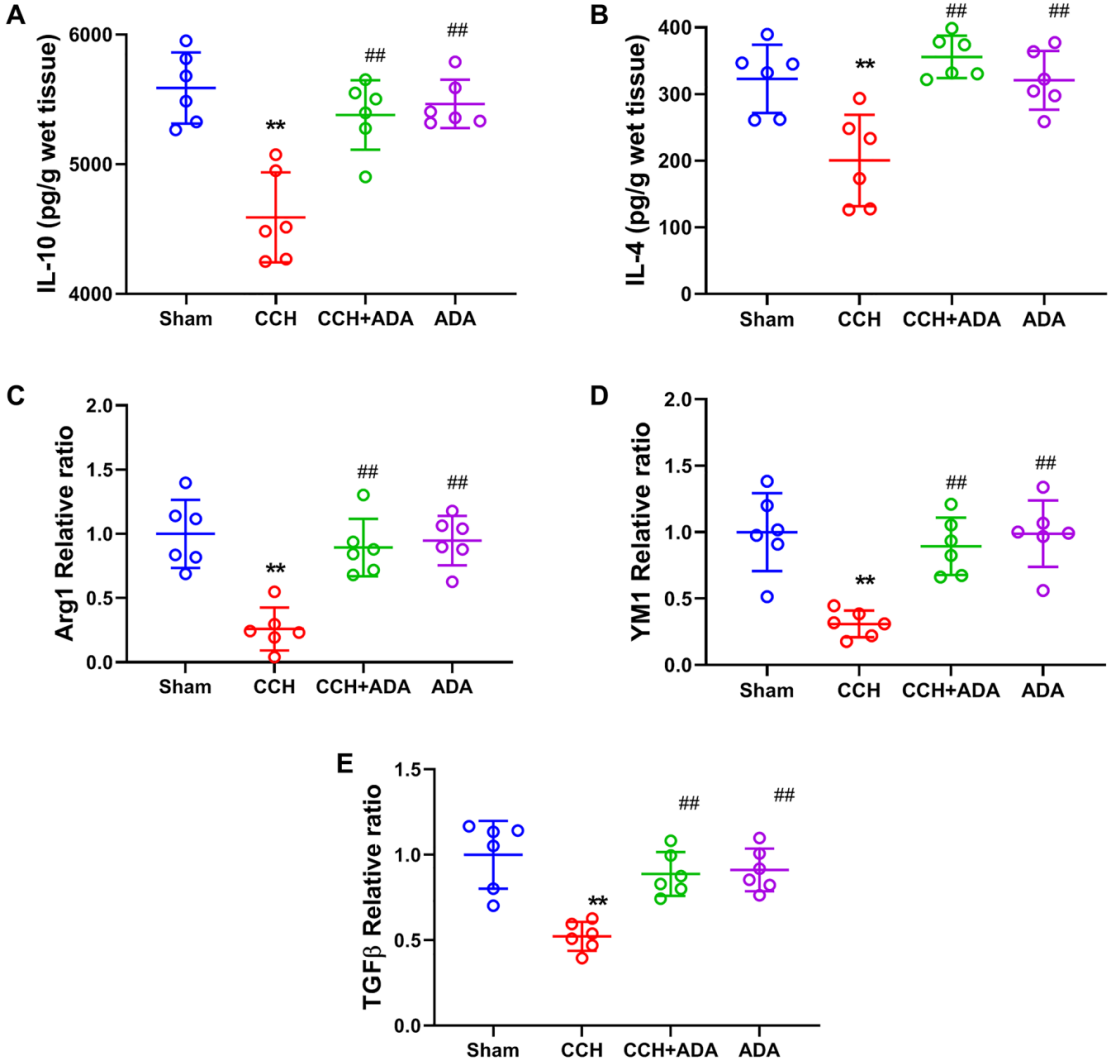
**ADA elevated the expression of M2 markers**. (**A**–**B**) The expression of the M2 markers IL-10 (A) and IL-4 (B) was evaluated by ELISA kits. (**C**–**E**) The mRNA levels of Arg1 (C), YM1 (D) and TGF-β (E) were evaluated by Q-PCR. All values are expressed as the mean ± SEM (*n* = 6). ^**^p < 0.01 vs. the sham group; ^##^*p* < 0.01 vs. the CCH group.

It is known that the polarization of microglia from the M2 to M1 phenotype promotes the activation of microglia and brain inflammation [21]. We therefore evaluated the polarization states of microglia upon different treatments by detecting known biomarkers for M1 and M2 microglia. We found that in CCH rats, the expression levels of the M1 markers TNFα, IL-12, IL-6, IFNγ and IL-1β were significantly upregulated. After treatment with ADA for two months, the expression of these M1 markers was reduced to normal levels (Figure 5A–5E). Moreover, CCH led to the suppression of the expression of IL-10 and IL-4 and the mRNA levels of Arg1, YM1 and TGFβ, which are well-known M2 markers. Importantly, ADA treatment restored the expression of these M2 markers to normal levels (Figure 6A–6E). Therefore, ADA treatment promotes microglial polarization to the M2 phenotype.

### Adalimumab inhibited NF-κB signaling in CCH rats

**Figure 7:**
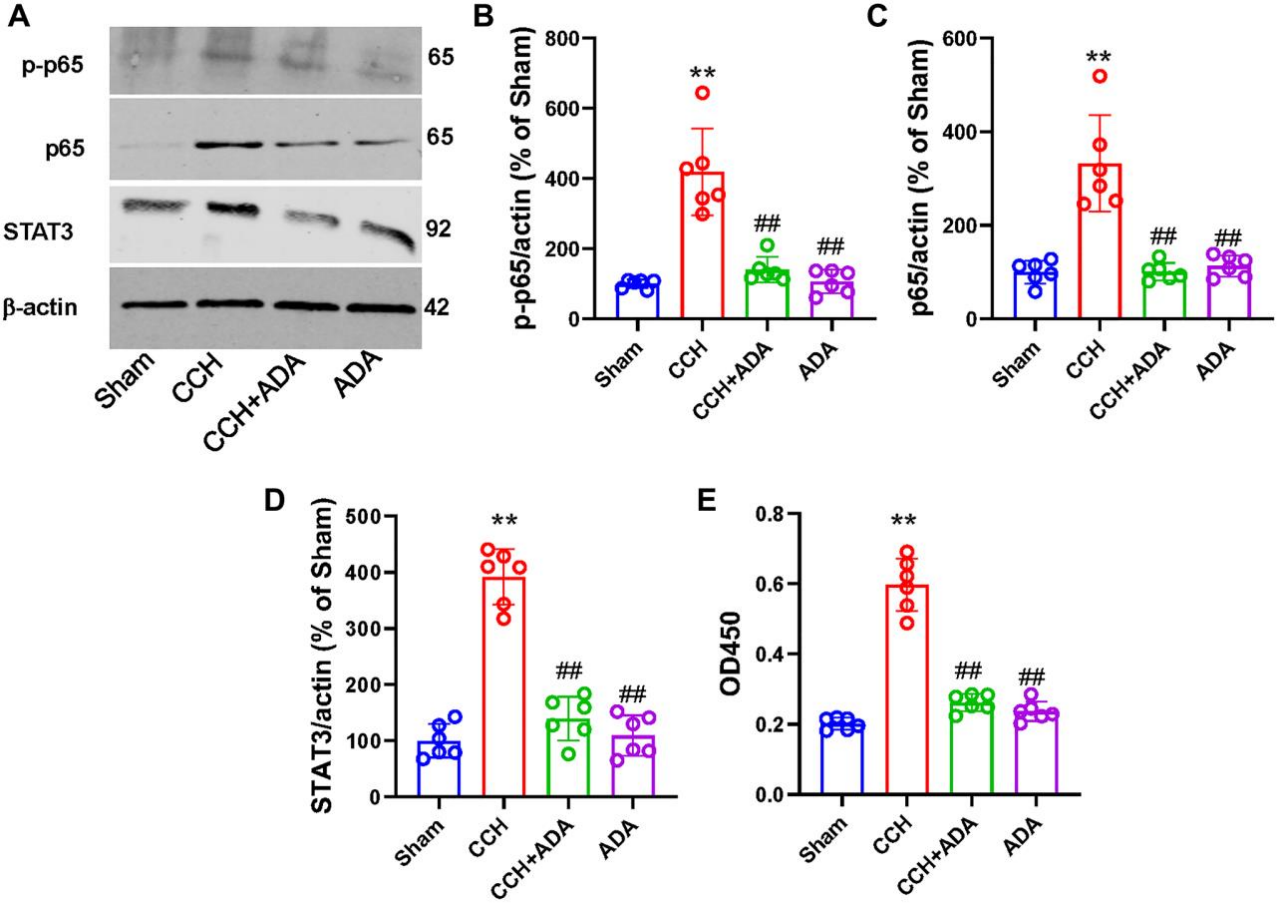
**ADA inhibited NF-κB signaling in CCH rats.** (**A**) Representative blots for p-p65, p65 and STAT3 in the different groups are shown. β-Actin was used as loading control. (**B**–**D**) Quantification of the optical intensity of p-p65 (B), p65 (C) and STAT3 (D). (**E**) NF-κB activity was evaluated by ELISA kits. All values are expressed as the mean ± SEM (*n* = 6). ^**^p < 0.01 vs. the sham group; ^##^*p* < 0.01 vs. the CCH group.

We then wanted to explore the potential mechanisms underlying the protective role of ADA in CCH. As the activation of NF-κB signaling has been identified as a key player in microglial activation and polarization [22], we assessed whether ADA could suppress NF-κB activity in CCH rats. Consistent with a previous study, the phosphorylation of the p65 subunit at the Ser536 site and the total expression of p65 and STAT3 were significantly increased in the hippocampi of CCH rats. However, ADA treatment effectively reduced the phosphorylation of p65 and the levels of p65 and STAT3 (Figure 7A–7D). Furthermore, ELISA revealed that NF-κB activity was increased in CCH rats but suppressed after ADA treatment (Figure 7E). No significant difference was found between the rats treated with ADA alone and the sham rats. These data suggested that ADA inhibits NF-κB signaling in CCH rats.

### Adalimumab protected against oxidative stress in CCH rats

**Figure 8:**
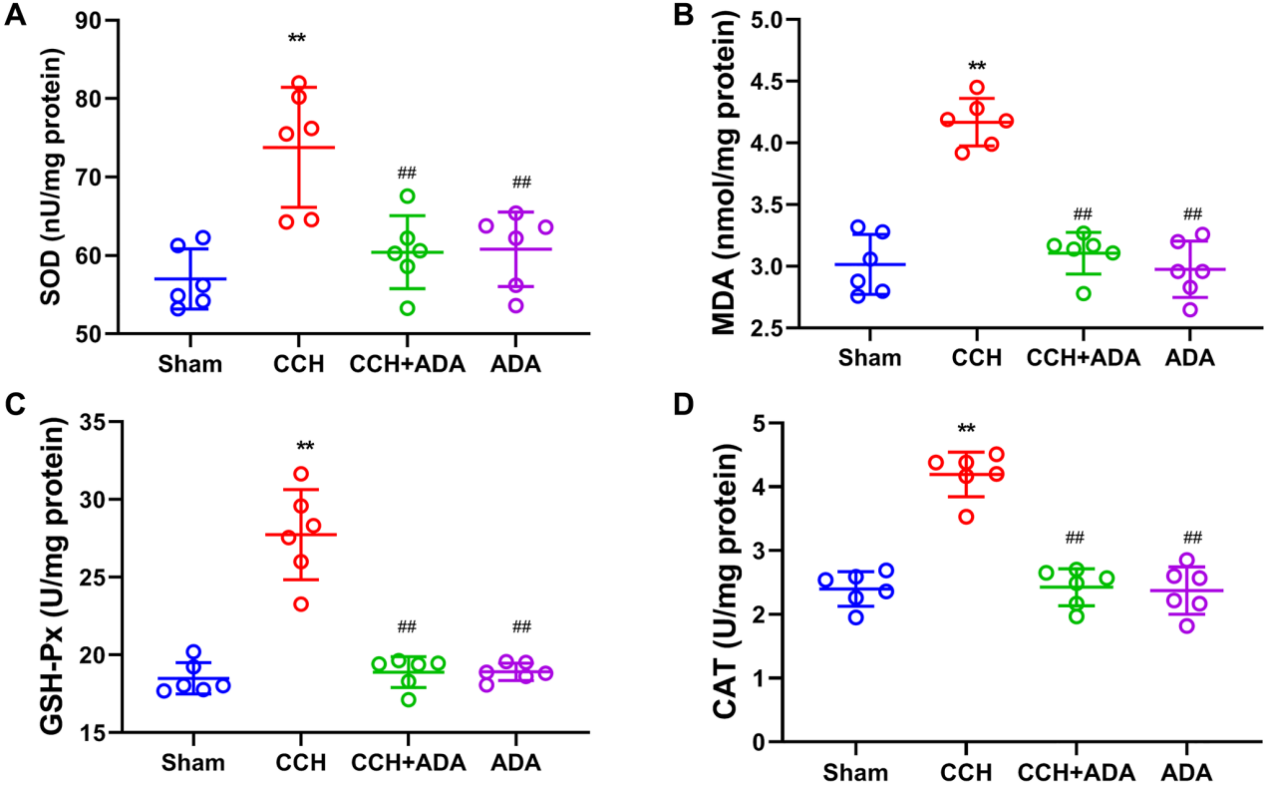
**ADA protected against oxidative stress in CCH rats.** The levels of SOD (**A**), MDA (**B**), GSH-Px (**C**) and CAT (**D**) in the different groups were measured by the commercial kits. All values are expressed as the mean ± SEM (*n* = 6). ^**^p < 0.01 vs. the sham group; ^##^*p* < 0.01 vs. the CCH group.

It is known that oxidative stress plays a central role in NF-κB signaling activation and that crosstalk between oxidative stress and NF-κB signaling is critical for microglial activation [23]. Thus, we evaluated the levels of SOD, MDA, GSH-Px and CAT by commercial kits. We found that CCH resulted in the elevation of SOD, GSH-Px, CAT and MDA levels, indicating an increase in lipid peroxidation and a compensatory increase in the levels of antioxidant enzymes. However, treating CCH rats with ADA significantly reduced oxidative stress (Figure 8A–8D). Therefore, administration of ADA could protect CCH rats from oxidative stress.

## DISCUSSION

Cognitive impairment is one of the key clinical manifestations of both vascular dementia and Alzheimer’s disease. It was previously reported that chronic cerebral hypoperfusion is a common underlying pathological change and contributes to cognitive decline in these diseases [24]. In both rodent models and human patients, neuroinflammation is widely observed in the brain [25, 26]. Previous studies have found that elevation of the levels of neuroinflammatory cytokines and enhanced gliosis can be found in the hippocampi of CCH rats [19]. Administration of luteolin, a polyphenolic compound that possesses anti-inflammatory effects, suppresses the activation of the NF-κB pathway and ameliorates cognitive impairment in CCH rats [27]. Huperzine A, a natural acetylcholinesterase (AChE) inhibitor, suppresses the overexpression of TNFα and protects against 2VO-induced memory impairment [28]. Duloxetine (DXT), a potent and balanced serotonin/norepinephrine reuptake inhibitor, is also able to reduce TNF-α and IL-1β levels in the hippocampal CA1 region in CCH rats [29]. The above evidence strongly suggests that targeting inflammatory responses might be a promising therapeutic strategy for memory impairments in CCH. In line with these data, we found that ADA, a commercially available anti-TNF-α drug, effectively inhibited neuroinflammation by suppressing the NF-κB signaling pathway. Furthermore, ADA treatment significantly reduced neuronal loss not only in our CCH model but also in a mouse model of retinal degeneration [30] and motor neuropathy in rheumatoid arthritis [31]. Importantly, ADA alleviated memory deficits in CCH, as shown by the Morris water maze test, further confirming the importance of anti-inflammatory responses in the treatment of memory decline caused by CCH.

It is known that microglia are the first line of defense in the brain against various pathological changes [32], and the activation of microglia plays an important role in the initiation, propagation and intensification of neuroinflammation in multiple neurological and psychiatric disorders [33, 34]. In CCH rats, a reduction in cerebral blood flow results in the activation of microglia, which in turn promotes the release of a large amount of neuroinflammatory factors and leads to the impairment of synaptic plasticity and cognitive functions [35]. Upon activation, microglia are generally polarized toward one of two phenotypes, M1 and M2, which contribute to neuroinflammatory responses [36]. The M1 phenotype is the classical activation phenotype, and M1 microglia promote the expression of proinflammatory molecules; however, the M2 phenotype is an alternative phenotype, and M2 microglia exert neuroprotective effects [37]. In the ischemic brain, M1 microglia facilitate secondary brain damage, while M2 microglia promote recovery following ischemia [38]. In the CCH brain, prolonged cerebral hypoperfusion is a major cause of microglial polarization to the M1 phenotype [39] but not the M2 phenotype. We observed here that ADA administration not only reduced the number of activated microglia but also promoted the switch of microglia from the M1 to the M2 phenotype. Previous studies suggested that M2 microglia play an essential role in mediating the regenerative response in demyelinating disorders by reinforcing the differentiation of oligodendrocytes [40], which has been implicated in the therapeutic effects of electroacupuncture on CCH-induced memory impairments [41]. Therefore, our proof-of-concept study provides experimental evidence for the potential role of ADA in promoting oligodendrocyte-associated regeneration in CCH.

Reactive oxygen species (ROS) are the key modulators of oxidative stress, which is caused by oxidant/antioxidant imbalance. Due to a reduction in cerebral blood flow, excessive ROS are produced in the brains of animals with CCH [18], leading to oxidative stress and the impairment of not only neurons but also vascular endothelial cells and glia [42]. In line with previous studies, we demonstrated here that elevation of the levels of both antioxidants and oxidants in the CCH brain indicates oxidative stress. It is known that oxidative stress and continuous hypoperfusion in the brain promote the expression of nitric oxide synthase (NOS) and further increase the levels of ROS and nitrogen oxides, thus forming a vicious cycle [42]. Importantly, ROS can oxidize proteins and lipids and then damage DNA, leading to further activation of microglia and astrocytes and thus the initiation of neuroinflammatory responses [43]. Furthermore, NF-κB is a redox-sensitive transcription factor that can enhance the transcription of proinflammatory cytokines and the activation of inflammasomes, all of which are related to neuronal injury, in CCH [44]. In addition, numerous studies have suggested that the cooperation of ROS with components of the inflammatory response plays important roles in the pathological changes in AD [45], which is the most prevalent neurodegenerative disorder in aged people and is strongly associated with brain hypoperfusion [46]. We also revealed that administration of ADA could restore the antioxidant/oxidant balance in the CCH brain and suppress the activity of NF-κB. It is known that NF-κB can regulate the transcription of multiple genes, including chemokines, cytokines, proinflammatory enzymes, adhesion molecules, and proinflammatory transcription factors. Most of these genes are important for neuroinflammation. Thus, ADA can inhibit neuroinflammation both directly and indirectly.

Taken together, we have first demonstrated that ADA supplementation is able to rescue neuronal damage, neuroinflammation, microglial activation, oxidative stress, and memory deficits in CCH.
